# The Muscle–Brain Axis in Aging: Mechanistic and Clinical Perspectives on Resistance Training and Cognitive Function

**DOI:** 10.3390/biology15020154

**Published:** 2026-01-15

**Authors:** Shuyun Yu, Yi Fan, Bochao You, Haoyue Zhang, Zhenghua Cai, Sai Zhang, Haili Tian

**Affiliations:** 1 School of Exercise and Health, Shanghai University of Sport, Shanghai 200438, China; 2421517015@sus.edu.cn (S.Y.); 2521517004@sus.edu.cn (Y.F.); 2421517013@sus.edu.cn (B.Y.); zhangdabai333@163.com (H.Z.); chasecome@163.com (Z.C.); 2 Beijing Research Institute of Sports Science, Beijing 100075, China

**Keywords:** cognitive impairment, resistance training, myokines, muscle–brain axis, older adults

## Abstract

The global aging population has led to a rising prevalence of cognitive impairment, posing a significant public health challenge. Resistance training (RT) is a non-pharmacological intervention that has been increasingly investigated for its potential to support cognitive function in older adults. Clinical evidence suggests that RT may be associated with benefits in certain cognitive domains, including memory, executive function, processing speed, and visuospatial ability. However, findings across studies remain heterogeneous, with several trials reporting neutral outcomes. Most intervention studies involve structured RT programs conducted at moderate to high intensity and performed multiple times per week. However, optimal training parameters have not yet been clearly established due to variability in study design and the absence of formal dose–response analyses. Emerging evidence suggests that the cognitive effects of RT may be mediated, at least in part, through muscle–brain axis signaling involving exercise-induced myokines. Factors such as irisin, brain-derived neurotrophic factor, interleukin-6, interleukin-15, and insulin-like growth factor-1 have been implicated in processes related to neuroplasticity, neuroinflammatory regulation, and neurovascular function, primarily based on preclinical and translational research. This review synthesizes current evidence on RT-related molecular mechanisms and clinical findings to provide an integrative perspective on the potential role of resistance training in mitigating age-related cognitive decline.

## Simple Summary

This review examines the potential benefits of resistance training on cognitive function in older adults. We synthesize evidence from clinical and mechanistic studies to investigate possible biological pathways underlying these benefits. Current evidence from intervention studies, typically involving 2–3 sessions per week, suggests that resistance training may stimulate the release of myokines into circulation. These myokines are hypothesized to reach the brain, where they could support neuronal health, promote synaptic plasticity, and modulate neuroinflammation. While the muscle–brain axis represents a promising mechanism, causality remains to be fully established, and further research is needed to confirm these pathways and optimize exercise prescriptions. Understanding these processes could inform non-pharmacological strategies to support brain health during the aging process.

## 1. Introduction

Cognitive impairment is a prevalent condition in older adults, characterized by a decline in one or more cognitive domains, including memory, language, visuospatial abilities, executive function, calculation, and judgment [1]. This decline is a hallmark of several neurodegenerative diseases, including Alzheimer’s disease (AD) and Parkinson’s disease (PD), as well as other forms of dementia [2]. Importantly, cognitive impairment can also occur in the absence of clear-cut dementia, often presenting in the form of mild cognitive impairment (MCI), which may progress to more severe forms of dementia, such as AD and vascular dementia, over time [3,4].

With the rapidly aging global population, the prevalence of cognitive impairment is increasing. According to projections by the World Health Organization (WHO), the number of individuals aged 60 or older is expected to rise from 1 billion in 2020 to 2.1 billion by 2050. Similarly, the population of individuals aged 80 and above is expected to triple, reaching 426 million by 2050 [5]. As a result, the burden of cognitive impairment and related neurodegenerative conditions, including dementia, is also rising. Currently, over 57 million people worldwide are living with dementia, with approximately 10 million new cases diagnosed annually [6]. Cognitive impairment not only severely impacts functional independence but also contributes to increased disability, mortality, and substantial socioeconomic costs. It has become a pressing public health issue in the 21st century, emphasizing the urgent need for effective prevention and intervention strategies.

Exercise, particularly resistance training (RT), has emerged as a promising non-pharmacological intervention for improving cognitive function, including learning, memory, and executive function. A growing body of research suggests that different types and intensities of exercise can produce varying effects on distinct cognitive domains, with RT showing notable benefits in individuals with MCI and dementia [7]. Notably, studies have shown that RT, especially when combined with other interventions, may slow cognitive decline and improve memory in people with cognitive impairment, including MCI [8]. However, the precise mechanisms through which RT influences cognitive function remain unclear. Recent evidence suggests that the muscle–brain axis plays a crucial role in mediating these effects, involving the release of myokines, neurotrophic factors, anti-inflammatory pathways, and metabolic regulation [9].

Therefore, this review aims to synthesize the current clinical and mechanistic evidence on how RT enhances cognitive function in older adults, with a particular focus on the muscle–brain axis. Our analysis proceeds in three stages: first, evaluating the cognitive benefits of RT in individuals with varying degrees of cognitive impairment; second, exploring the epidemiological and interventional links between muscle function and cognition; and third, providing a detailed examination of the specific myokine-mediated mechanisms (such as Irisin, BDNF, and IGF-1) that underlie these cognitive benefits. By elucidating these mechanisms, this review aims to provide a framework for optimizing RT interventions and informing future research on muscle–brain communication.

## 2. Methods

For this narrative review, a comprehensive literature search was conducted to explore the relationship between resistance training and cognitive function in older adults. The primary databases utilized were PubMed, Web of Science, and Google Scholar, with the search executed from January 2015 to October 2025. The search strategy incorporated the following keywords: “resistance training”, “cognitive impairment”, “dementia”, “myokines”, “muscle–brain axis”, “older adults”, and “aging”.

The selection criteria were designed to focus exclusively on studies investigating the effects of resistance training on cognitive outcomes or the associated myokine-mediated pathways in individuals aged 60 years and older. Eligible studies included human randomized controlled trials, longitudinal studies, and relevant mechanistic reviews. Seminal animal studies were also included where necessary to provide insights into key underlying mechanisms.

Studies were excluded if they concentrated solely on aerobic exercise or if they involved neurological disorders where cognitive decline was not the principal feature, such as isolated stroke or traumatic brain injury. Additionally, relevant literature was identified through manual screening of the reference lists of selected key articles, with particular attention given to research elucidating the roles of myokines in mediating cognitive benefits.

## 3. The Impact of RT on Cognitive Impairment in Older Adults

Cognitive function encompasses higher-order mental processes such as attention, working memory, and executive control that support adaptive behavior. Aging-related cognitive decline is commonly associated with gradual alterations in brain structure and function, including reduced synaptic plasticity, disrupted large-scale functional connectivity, and compromised white matter integrity [10,11]. Cognitive impairment in older adults is increasingly conceptualized as a continuum, ranging from regular age-related changes to subjective cognitive decline, MCI, and ultimately neurodegenerative diseases such as AD [12,13]. These stages reflect overlapping but heterogeneous neurobiological processes rather than discrete, stage-specific mechanisms. For instance, MCI has been linked to synaptic dysfunction, network-level dysconnectivity, and metabolic alterations, with growing evidence suggesting a potential role for gut–brain axis signaling and neuroinflammation. However, these mechanisms are neither universal nor exclusive [13,14]. In established neurodegenerative disorders, distinct proteinopathies—such as β-amyloid and tau accumulation in AD and α-synuclein aggregation in PD—contribute to disease-specific cognitive trajectories [15,16]. Given this neuropathological heterogeneity, the cognitive effects of RT may vary according to baseline cognitive status and neural integrity. Accordingly, this section examines RT-related cognitive outcomes across elderly populations categorized as cognitively healthy older adults, individuals with MCI, those with dementia, and patients with PD, enabling structured comparisons in commonly assessed domains (e.g., executive function and working memory) while acknowledging differences in underlying disease mechanisms. The characteristics of the included studies, along with their classification by cognitive status, are summarized in Table 1.

### 3.1. Naturally Aging Older Adult Population

During natural aging, older adults often experience a decline in both physiological and neurocognitive functions, which manifests as impairments in memory, executive function, and processing speed [28]. Multiple clinical studies have confirmed that RT can improve cognitive function and mitigate specific cognitive declines in healthy older adults through various mechanisms. For instance, Fragala et al. [17] reported that a six-week multi-joint RT intervention improved spatial perception by 40%, and reduced visual and physical reaction times by 14.6% and 14.0%, respectively, in adults around 70 years old. Additionally, RT programs that incorporate neuromuscular challenges, such as unstable surfaces or free weights, appear to be more effective than machine-based RT in enhancing higher-order cognitive functions.

Eckardt et al. [19] compared the effects of unstable versus stable RT in older adults aged 65–79 years. They found that 10 weeks of unstable free-weight training resulted in significant improvements in working memory (11% improvement in the Digit Memory Test, d = 0.32), processing speed (19% improvement in the Digit Symbol Substitution Test, d = 0.73), and response inhibition (8% improvement in the Stroop Colour-Word Test, d = 0.55), compared to stable machine-based RT, which showed minimal gains. These cognitive benefits are likely due to enhanced prefrontal-cerebellar connectivity, which is stimulated by the dynamic balance and sensorimotor demands of instability training. This type of training likely enhances neural efficiency and plasticity in circuits that support motor control and executive functions.

Further evidence of RT’s benefits comes from Coetsee et al. [18], who observed that after 16 weeks of RT, older adults showed a 27.35% improvement in reaction time on the Stroop task, outperforming both high-intensity interval training (17.80%, ES = 0.79) and control groups (24.83%, ES = 0.94). Additionally, accuracy increased by 2.41%. These results suggest that RT may enhance executive cognition, potentially by strengthening inhibitory control mediated by the anterior cingulate cortex, a region essential for conflict monitoring and cognitive control during response inhibition tasks.

It is crucial to note that the combination of exercise modalities and sex differences has a significant impact on the efficacy of interventions. A systematic review and meta-analysis by Barha et al. [29] emphasized that RT has a notably greater effect on improving executive functions in women compared to men. Specifically, when the proportion of female participants in studies was higher than 71%, the effect size for RT on executive functions was significantly larger (Hedges’ g = 0.639) compared to studies with fewer female participants (≤71%), where the effect size was 0.150. This suggests that women tend to benefit more from RT in terms of cognitive performance, particularly in executive function, than men. Moreover, multimodal training, which combines both aerobic and resistance training, was found to lead to greater improvements in overall cognitive function and explicit memory compared to single modality RT, with an effect size of 1.801 for global cognitive function.

Collectively, RT can improve executive function, processing speed, and spatial perception in healthy older adults through various mechanisms, including the activation of neuromuscular regulation and the enhancement of functional connectivity in brain regions. However, the effects of RT are influenced by exercise mode design and gender differences, which lay the groundwork for personalized cognitive health interventions for the aging population.

### 3.2. Older Adult Population with Mild Cognitive Impairment

Mild cognitive impairment (MCI) represents a transitional stage between normal aging and dementia, characterized by measurable cognitive decline without substantial impairment of daily functioning [3]. Accumulating evidence from randomized controlled trials suggests that RT can effectively improve cognitive performance in older adults with MCI.

Six months of high-intensity progressive resistance training (PRT) has been shown to significantly enhance global cognitive function in individuals with MCI (F(90) = 4.1, *p* < 0.05). These cognitive gains were accompanied by attenuated structural decline in the posterior cingulate cortex, with an annualized increase in gray matter thickness (+0.01 mm) compared with an annualized reduction observed in non-exercising controls (−0.05 mm) [30]. Notably, an 18-month follow-up study demonstrated that PRT not only sustained cognitive improvements but also preserved the structural integrity of hippocampal subregions, which are particularly vulnerable to Alzheimer’s disease pathology [20].

Interventions that increase cognitive demands during RT may further enhance cognitive outcomes. In a single-masked randomized controlled trial, Baek et al. [21] reported that older adults with cognitive impairment who completed six weeks of dual-task RT exhibited greater improvements in Mini-Mental State Examination scores than those performing single-task RT (mean increases of 2.22 vs. 1.59 points). Similarly, Vints et al. [22] found that a 12-week RT program improved inhibitory control, as reflected by reduced reaction times on a Go/No-Go task, particularly in older adults at high risk of MCI. Within the RT group, increases in circulating insulin-like growth factor-1 were associated with faster processing speed, suggesting a potential neurotrophic contribution to the cognitive benefits associated with RT.

Collectively, RT interventions of varying duration and complexity consistently improve cognitive function in individuals with MCI, with particularly robust effects on executive control. These benefits appear to be supported by sustained cognitive gains, concurrent preservation of vulnerable brain structures, and favorable neurotrophic adaptations, highlighting RT as a promising non-pharmacological strategy for mitigating cognitive decline in high-risk aging populations.

### 3.3. Older Adults with Dementia

Dementia is a progressive neurodegenerative condition characterized by global cognitive decline and functional impairment, with pathological processes involving β-amyloid accumulation, tau hyperphosphorylation, and chronic neuroinflammation [31,32]. In recent years, increasing attention has been given to exercise-based interventions, particularly RT, as potential non-pharmacological strategies to mitigate cognitive deterioration in this population.

Evidence from systematic reviews suggests that RT may confer cognitive benefits in individuals with dementia. For example, Huang et al. [8] reported that RT demonstrated larger effect sizes than other exercise modalities in slowing cognitive decline, particularly for global cognition (SMD = 1.05, 95% CI: 0.56–1.54), executive function (SMD = 0.85, 95% CI: 0.21–1.49), and memory (SMD = 0.32, 95% CI: 0.01–0.63). These findings indicate a potential advantage of RT, although the heterogeneity of included studies warrants cautious interpretation.

At the individual trial level, Liu et al. [23] conducted a single-blind randomized controlled trial in older adults with dementia and demonstrated that four weeks of RT significantly improved MMSE scores (*p* = 0.014), accompanied by a reduction in plasma monocyte chemotactic protein-1 (MCP-1), a pro-inflammatory marker. While this biomarker change suggests a possible link between RT and modulation of systemic inflammation, the causal role of inflammatory pathways in mediating cognitive improvements remains to be clarified.

In contrast, findings from large pragmatic trials suggest that the effects of exercise on dementia are not uniformly positive. The DAPA trial by Lamb et al. [24], which enrolled 494 individuals with mild to moderate dementia, showed that a four-month combined aerobic and resistance training program was associated with a modest worsening in cognitive performance, as measured by the ADAS-Cog score (between-group difference −1.4 points, 95% CI: −2.6 to −0.2; *p* = 0.03), compared with usual care. These results highlight the potential influence of dementia subtype heterogeneity, intervention intensity, and training duration on cognitive outcomes.

Collectively, current evidence indicates that the cognitive effects of RT in individuals with dementia are variable. While short-term and targeted RT interventions may yield modest cognitive benefits, particularly in relation to inflammatory modulation, large-scale trials suggest that exercise may not uniformly slow cognitive decline in established dementia. These discrepancies underscore the importance of individualized exercise prescription and the need for further mechanistic studies to clarify which patient subgroups are most likely to benefit.

### 3.4. Older Adults with Parkinson’s Disease

Parkinson’s disease (PD) is a progressive neurodegenerative disorder characterized by the degeneration of dopaminergic neurons in the substantia nigra, leading to both motor dysfunction and non-motor symptoms, including cognitive impairment [33]. Accumulating evidence from randomized controlled trials indicates that RT, particularly when designed with progressive loading or increased motor complexity, can yield meaningful cognitive benefits in individuals with PD.

In a controlled trial, David et al. [25] compared progressive and non-progressive RT over 24 months and reported that participants undergoing progressive RT demonstrated superior improvements in working memory, inhibitory control, and attentional performance. These findings suggest that sustained increases in training load may be associated with enhanced cognitive outcomes, although the underlying neural mechanisms were not directly assessed.

Consistent with these observations, Silva-Batista and colleagues [26] showed that 12 weeks of unstable RT significantly improved global cognition in patients with moderate to severe PD, as reflected by a mean increase of approximately 6 points in the Montreal Cognitive Assessment (MoCA) score. Improvements were observed across multiple cognitive domains, including visuospatial–executive function, attention, abstract reasoning, delayed recall, and orientation. Notably, a subsequent randomized trial focusing on PD patients with freezing of gait (PD + FOG) demonstrated that adaptive unstable RT not only enhanced frontal lobe function but also accounted for 43% of the variance in the reduction in freezing severity [27]. These findings suggest a strong correlation between improvements in executive control and motor symptoms in this population.

Although the precise neurobiological mechanisms remain to be fully elucidated, it has been proposed that high-complexity RT paradigms, such as unstable or coordinative training, may place greater demands on executive and attentional control, thereby facilitating cognitive–motor interactions. This interpretation is consistent with the comorbidity loop hypothesis proposed by Intzandt et al. [34], which suggests that exercise-induced adaptations within cortical–striatal networks may contribute to concurrent improvements in cognitive and motor function.

Collectively, available clinical evidence suggests that while traditional RT may confer limited cognitive benefits in PD, RT protocols incorporating progressive overload or increased motor complexity—such as unstable RT—are more likely to produce meaningful improvements in executive function, attention, and global cognition. Long-term progressive RT appears to offer moderate benefits for attentional and working memory processes. In contrast, short-term high-complexity RT may be particularly advantageous for individuals with advanced disease or freezing of gait.

Overall, the cognitive effects of RT in older adults appear to be heterogeneous and influenced by baseline cognitive status, disease condition, and training characteristics. Evidence suggests that RT incorporating progressive overload or increased motor and task complexity is more consistently associated with cognitive benefits than low-complexity or non-progressive protocols. Across studies, interventions linked to cognitive improvements commonly involve training frequencies of two to three sessions per week and durations ranging from several weeks to long-term programs, indicating that sustained engagement may be an essential factor. In addition, emerging evidence points to a non-linear relationship between RT dose and cognitive outcomes, highlighting the potential importance of individualized program design rather than a uniform prescription. Together, these findings support RT as a promising, yet context-dependent, non-pharmacological approach for maintaining or improving cognitive function in aging populations.

## 4. The Association Between Muscle Mass or Function and Cognitive Impairment in Older Adults

### 4.1. Cross-Sectional Evidence

Multiple epidemiological studies focusing on middle-aged and older adults have consistently identified a strong link between muscle mass or function and cognitive performance across various regions and health conditions (Table 2). For instance, Wang et al. [35] examined the body composition of 2540 elderly individuals and found that the Digit Symbol Substitution Test (DSST) scores of the low muscle mass group were significantly lower than those of the normal muscle mass group—for example, 43.56 ± 18.36 vs. 47.56 ± 17.44 (*p* < 0.001) when defined by appendicular lean mass (ALM). This negative correlation was consistent across the age subgroups of 60–69, 70–79, and 80 and older, with strong associations observed in the ALM/BMI-defined low muscle mass category (*p* < 0.001 for both 60–69 and 70–79 years; *p* = 0.009 for those 80 years and older). Furthermore, higher muscle strength, measured by peak force, was also associated with better cognitive function. Jiang et al. [36] analyzed data from over 40,000 participants in the UK Biobank and showed that greater grip strength was positively linked to multiple cognitive domains, including faster reaction time (t = −19.56, FDR-corrected *p* = 2.42 × 10^−83^), higher fluid intelligence (β = 0.019, FDR-corrected *p* = 0.041), and better prospective memory (OR = 1.27, FDR-corrected *p* = 3.75 × 10^−23^). Furthermore, Liao et al.’s [37] study of 245 elderly individuals demonstrated that both grip strength and lower limb strength, as assessed by the 30 s sit-to-stand test, are significantly associated with working memory performance. This is evidenced by positive correlations with 1-back and 2-back accuracy (r = 0.342 and r = 0.215 for grip strength; r = 0.616 and r = 0.441 for lower limb strength, all *p* < 0.001). Structural equation modeling further confirmed that both measures of muscle strength positively predicted accuracy in the 1-back and 2-back tasks, emphasizing a strong connection between muscle function and cognitive performance in older adults. Notably, a study combining cross-sectional and Mendelian randomization methods confirmed a bidirectional causal relationship between grip strength and cognitive impairment [38]. Although these findings are consistent with causal inference, residual confounding and reverse causality cannot be ruled out; therefore, the bidirectional association should be interpreted with caution. Low grip strength is associated with a higher risk of cognitive impairment, and cognitive impairment also accelerates the decline in grip strength. Further research, employing more robust methodologies, including longitudinal studies, would be necessary to confirm these associations and better understand their causal nature.

Further research indicates that muscle strength plays a significant mediating role in the relationship between physical activity and cognitive function. A cross-sectional study by Chen et al. [39] found that lower limb muscle strength accounted for 20.1% of this association. Additionally, Li et al.’s [40] study revealed significant correlations among physical activity, muscle strength, working memory, and cognitive function in older adults, supported by strong statistical evidence. Specifically, physical activity level showed a strong positive correlation with grip strength (r = 0.559, *p* < 0.001), which was also positively linked to 1-back accuracy (r = 0.417, *p* < 0.001) and negatively related to response times in working memory tasks. Furthermore, 1-back accuracy alone explained 11.6% of the variance in cognitive function scores (R^2^ = 0.116, *p* < 0.001). The authors suggested a biochemical cascade: physical activity → muscle strength → working memory → cognitive function, shedding light on potential mechanisms by which exercise interventions may improve cognitive performance. This model offers a more comprehensive theoretical framework for future research in the field.

### 4.2. Longitudinal and Interventional Evidence

Prospective studies have further explored the temporal associations and potential mechanisms linking muscle mass or function to cognitive decline. Numerous cohort studies and randomized controlled trials have demonstrated that both overall and targeted muscle strength are associated with cognitive outcomes. However, these effects appear to vary across populations with different clinical characteristics.

A cohort study involving 1978 older Chinese adults [41], followed for seven years, demonstrated a significant association between low muscle mass and a decline in multidimensional cognitive scores (β = −0.14, *p* = 0.002). This relationship was consistent across gender subgroups, suggesting that whole-body muscle mass may serve as an early indicator of cognitive decline. Similarly, in a large-scale prospective study of Chinese middle-aged and older adults, Liu et al. [42] tracked 9333 participants aged 45 and older over four years and found that higher baseline grip strength was associated with better overall cognitive performance and a slower rate of cognitive decline, indicating a potential protective role of upper limb muscle strength.

In contrast, evidence from interventional studies has primarily been derived from specific clinical subgroups. Zhao et al. [43] conducted a randomized controlled trial involving 103 older adults with type 2 diabetes and demonstrated that 12 months of high-intensity power training significantly improved lower limb muscle strength. Specifically, the increase in knee extension strength was notably linked to improved executive function, as measured by the Trails B-A test (r = −0.41, *p* = 0.02). These findings suggest that resistance training–induced cognitive benefits may be particularly relevant for older adults with metabolic disorders. Additionally, a toe-grip training intervention in frail older adults demonstrated that improvements in lower-limb small muscle group strength were positively correlated with changes in MMSE score (r = 0.415, *p* = 0.013) [44], highlighting the potential cognitive relevance of localized neuromuscular adaptations within a frailty context.

Notably, Mavros et al. [45] reported in the SMART trial that progressive resistance training enhanced global cognitive function in patients with MCI, with increases in muscle strength—rather than aerobic capacity—acting as a key mediating factor. Taken together, these studies indicate that the cognitive effects of resistance-based interventions may be population-specific and influenced by underlying health status. Therefore, caution is warranted when extrapolating findings across clinically distinct groups, and conclusions should be interpreted within their respective clinical contexts.

## 5. The Mechanisms Underlying the Improvement of Cognitive Function by RT: Myokine-Mediated Muscle–Brain Axis

Skeletal muscle is not only the primary organ of the human musculoskeletal system but also a crucial regulatory tissue with endocrine functions. The concept of ‘myokines’ was proposed by Pedersen et al. [46], has significantly enhanced our understanding of the bidirectional communication between skeletal muscle and other organs. Myokines are bioactive peptides released during muscle contraction that can regulate the functions of various organ systems, including the brain, through autocrine, paracrine, or endocrine pathways [47]. To date, more than 600 muscle-derived proteins have been implicated in systemic regulation [48].

Given the heterogeneity of myokines and the varying strength of the available evidence, this review does not aim to provide an exhaustive classification of myokines. Instead, five myokines—Irisin, BDNF, IL-6, IL-15, and IGF-1—were pragmatically selected for focused discussion based on their frequent reporting in the exercise–cognition literature, relatively well-characterized biological mechanisms, and potential relevance to cognitive outcomes in aging populations. It should be noted that the level of evidence supporting these myokines spans preclinical, translational, and limited clinical studies, and the degree of mechanistic certainty therefore varies across factors.

**Figure 1:**
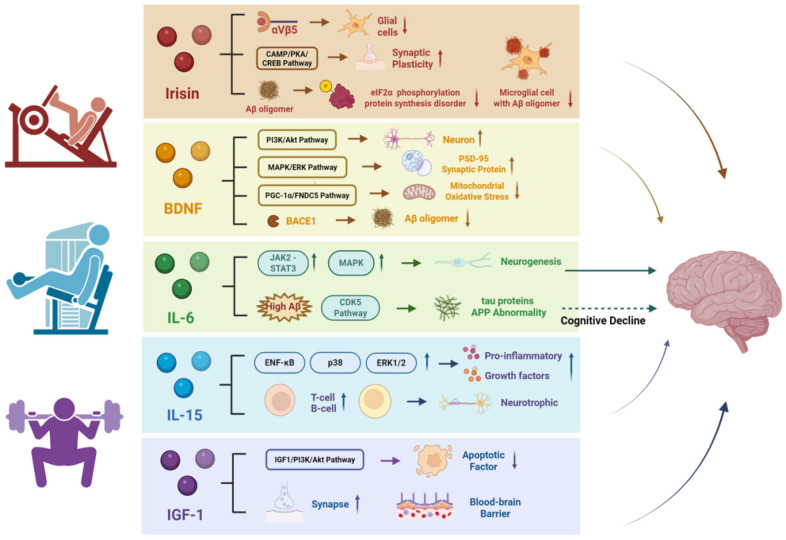
Potential mechanisms underlying myokines’ effects on cognitive function. Horizontal arrows indicate the direction of signaling pathways or mechanistic processes linking myokines to downstream molecular and cellular effects. Vertical arrows denote the direction of change, with upward arrows (↑) indicating upregulation or enhancement and downward arrows (↓) indicating downregulation or inhibition. Arrows pointing toward the brain represent the potential influence of these myokine-mediated mechanisms on brain function and cognitive outcomes.

Other candidate myokines, such as cathepsin B (CTSB), have been shown to promote BDNF expression in animal models [49]. However, evidence supporting CTSB as a biomarker or mediator of exercise-related cognitive improvement in humans remains preliminary and indirect [50]. Similarly, factors such as FGF-21 appear to be more strongly associated with metabolic regulation, and their potential influence on cognitive function requires further validation. Accordingly, the following sections will discuss key myokines with careful consideration of the experimental context and evidence level, distinguishing between findings derived from animal models and those supported by human studies (Figure 1).

### 5.1. Irisin

As an exercise-induced myokine, Irisin was initially identified as being associated with the development of brown fat and the regulation of energy metabolism [51]. Evidence from both animal and human studies indicates that exercise can increase circulating Irisin levels, although the magnitude and consistency of this response vary across experimental contexts. In mice, three weeks of voluntary wheel running increased plasma Irisin levels by approximately 65%. In human studies, 10 weeks of supervised endurance training in healthy adults was associated with a twofold increase in circulating Irisin compared with non-exercise conditions. Furthermore, Guo et al. [52] reported that regular resistance training significantly elevated Irisin levels in humans, with a circadian pattern peaking around 6 PM.

At the molecular level, exercise activates the PGC-1α signaling pathway in skeletal muscle, inducing the expression of the FNDC5 gene, which is subsequently cleaved to release Irisin into the circulation [53]. While alterations in the FNDC5/Irisin axis have been implicated in AD, it remains unclear whether the observed effects are mediated by cleaved Irisin or by the full-length, membrane-bound FNDC5 protein, particularly in human pathophysiology. Most mechanistic evidence linking Irisin to brain function is derived from preclinical studies. Christiane D. Wrann’s research team [54] demonstrated in mouse models that peripherally administered Irisin suppressed hippocampal astrocyte and microglial activation through binding to the αVβ5 integrin receptor complex, leading to reduced neuroinflammatory signaling and improved cognitive performance in Alzheimer’s disease models. These findings support a mechanistic role for Irisin in modulating neuroinflammation in animal models, a process that is broadly implicated in neurodegeneration.

Similarly, Lourenço et al. [55] found that Irisin enhances cognitive function through multiple mechanisms. In Alzheimer’s disease models—specifically, transgenic APP/PS1 mice and Aβ oligomer-treated neuronal cultures—Irisin has been shown to improve synaptic plasticity by activating the hippocampal cAMP/PKA/CREB signaling pathway, thereby rescuing impaired long-term potentiation [56,57]. At the same time, it prevents Aβ oligomer-induced phosphorylation of eIF2α and issues with protein synthesis, thereby reducing endoplasmic reticulum stress in neurons [58,59]. Additional preclinical evidence suggests that Irisin reduces Aβ–neuron binding, preserves dendritic spine density, and regulates synaptic gene expression, including Egr1 and Gria2 [55]. Although peripheral Irisin has been shown to cross the blood–brain barrier or indirectly elevate central Irisin levels in animal models, direct clinical evidence supporting these mechanisms in humans remains limited. Accordingly, current data primarily provide preclinical and translational support for Irisin as a mediator of exercise-induced neuroprotection, and its therapeutic relevance for human neurodegenerative diseases requires further clinical validation.

### 5.2. Brain-Derived Neurotrophic Factor (BDNF)

BDNF is a neurotrophic factor widely expressed in the nervous system, playing a critical role in neuronal survival, growth, differentiation, and synaptic plasticity [60]. Clinical evidence supports the responsiveness of BDNF to resistance exercise in older adults. Setayesh et al. [61] conducted a systematic review and meta-analysis of 11 randomized controlled trials and reported that regular RT significantly increased circulating BDNF levels in older adults, with a mean difference of 0.73 ng/mL (95% CI [0.04, 1.42]; *p* = 0.04).

The mechanistic pathways through which BDNF influences cognitive function have been primarily elucidated in preclinical models. At the cellular level, BDNF has been shown to enhance synaptic function by activating the PI3K/Akt pathway downstream of the TrkB receptor, thereby supporting neuronal survival [62]. Additionally, animal and in vitro studies have demonstrated that BDNF increases the expression of the synaptic protein PSD-95 through the MAPK/ERK pathway, thereby contributing to enhanced long-term potentiation [63].

BDNF has also been shown in rodent models to promote hippocampal neurogenesis by increasing the number of immature granule neurons [64] and by inducing transcription from BDNF exon IV, which enhances dendritic complexity [65]. Furthermore, preclinical evidence suggests that BDNF may modulate Alzheimer’s disease–related pathology by inhibiting β-secretase (BACE1) activity, thereby reducing Aβ deposition [66], and by attenuating mitochondrial oxidative stress through the PGC-1α/FNDC5 signaling pathway [67]. Although these mechanisms provide strong biological plausibility for the cognitive benefits of exercise-induced BDNF, direct evidence linking these pathways to cognitive outcomes in humans remains limited.

### 5.3. Interleukin-6 (IL-6)

Interleukin-6 (IL-6) is a pleiotropic cytokine involved in immune regulation, inflammatory responses, and neurobiological processes [68]. It is produced by multiple tissues, including contracting skeletal muscle during exercise and immune cells under inflammatory conditions. In humans, circulating IL-6 levels typically rise acutely in response to exercise, whereas chronically elevated IL-6 concentrations are more commonly associated with systemic inflammation and adverse health outcomes [69].

The relationship between IL-6 and cognitive function is highly context-dependent and remains incompletely understood. Observational evidence from longitudinal human cohort studies suggests that persistently elevated peripheral IL-6 levels are associated with poorer cognitive performance and an increased risk of cognitive decline, indicating that chronic low-grade inflammation may have detrimental effects on learning and memory [70]. Consistent with this, higher IL-6 concentrations have been linked to structural and functional brain alterations in aging and clinical populations, although these associations are primarily correlational in nature [71].

In contrast, interventional studies in humans suggest that exercise-induced modulation of IL-6 may contribute to a transient anti-inflammatory milieu rather than sustained neuroinflammation. For example, reductions in circulating IL-6 following structured exercise interventions have been associated with improvements in hippocampal volume and cognitive performance in older adults, indicating a potential indirect link between exercise-related IL-6 dynamics and cognitive outcomes [72]. However, direct mechanistic evidence demonstrating how IL-6 mediates cognitive changes in humans remains limited, and most human studies rely on peripheral biomarkers rather than direct measures of central nervous system signaling.

Mechanistic insights are therefore largely derived from preclinical animal and cellular models. At the preclinical level, IL-6 signaling has been shown to involve the JAK/STAT and MAPK pathways, which may contribute to its context-dependent effects on neuroinflammation and synaptic function [73]. Experimental studies in rodents suggest that acute or tightly regulated IL-6 signaling may promote non-amyloidogenic processing of APP and modulate microglial activity. In contrast, sustained IL-6 activation under pathological conditions can exacerbate tau phosphorylation and amyloid pathology [74,75]. These findings underscore the dualistic nature of IL-6 signaling and highlight that its neurobiological effects depend on source, timing, and inflammatory context.

Taken together, current evidence supports a nuanced interpretation in which IL-6 may exert either adaptive or maladaptive effects on cognitive function, depending on whether physiological or pathological conditions prevail. While preclinical data provide crucial mechanistic plausibility, further translational and clinical studies are required to clarify the causal role of IL-6 in human cognitive aging and neurodegenerative disease.

### 5.4. Interleukin-15 (IL-15)

Interleukin-15 (IL-15) is a cytokine abundantly expressed in skeletal muscle and is recognized as an exercise-responsive myokine with critical immunoregulatory functions [76]. Exercise-induced activation of the muscular p38γ signaling pathway has been shown to increase circulating IL-15 levels, supporting its role in muscle–immune communication in humans [76]. However, direct evidence linking exercise-related changes in IL-15 to cognitive outcomes in humans remains limited, and the current understanding of its neurobiological effects is primarily derived from preclinical research [77].

IL-15 is a multifunctional immune-inflammatory mediator whose biological effects appear to be highly context dependent. Experimental studies indicate that dysregulated or sustained IL-15 expression can activate the NF-κB, p38 MAPK, and ERK1/2 signaling pathways, thereby promoting the production of pro-inflammatory cytokines and potentially contributing to neural dysfunction [70,78]. At the same time, preclinical animal and cellular studies suggest that IL-15 may exert neurotrophic, anti-apoptotic, and neurogenic effects under specific physiological or compensatory conditions, partly through its regulation of T- and B-lymphocyte activity and neuroimmune crosstalk [79]. These seemingly divergent effects highlight that the impact of IL-15 on the central nervous system is strongly influenced by its concentration, cellular source, and inflammatory milieu, a pattern consistent with other cytokines involved in neuroinflammation.

Human observational evidence further underscores this complexity. Compared with healthy individuals and patients with vascular dementia, patients with Alzheimer’s disease exhibit lower circulating IL-15 levels in peripheral blood [80]. In contrast, elevated IL-15 concentrations have been reported in the cerebrospinal fluid of individuals with AD and frontotemporal dementia relative to patients with non-inflammatory neurological conditions [81,82]. This apparent dissociation between peripheral and central IL-15 levels may reflect differences in cytokine transport, local production, or regulation of the blood–brain barrier, and cautions against oversimplified interpretations based on single-compartment measurements [83].

Collectively, current evidence suggests that IL-15 may play a role in neuroimmune processes relevant to cognitive decline; however, its role remains largely inferential and is predominantly supported by preclinical data. While alterations in IL-15 signaling have been proposed as potential biomarkers or modulators of neurodegenerative pathology [79,84], further translational and longitudinal clinical studies are required to determine whether IL-15 plays a causal, compensatory, or secondary role in human cognitive aging and dementia.

### 5.5. Insulin-like Growth Factor 1 (IGF-1)

Insulin-like growth factor 1 (IGF-1) is a neurotrophic factor that plays essential roles in neuronal growth, differentiation, and survival during brain development and aging [85]. Although the liver is considered the primary source of circulating IGF-1, accumulating evidence suggests that contracting skeletal muscle also releases IGF-1, and that exercise training can modulate circulating IGF-1 levels [86,87]. In a human randomized intervention study, Chen et al. [88] reported that eight weeks of resistance training significantly increased serum IGF-1 levels (+0.16 ng/mL) in older adults with sarcopenic obesity. While cognitive outcomes were not directly assessed, these findings support a potential role for exercise-induced IGF-1 in muscle–brain communication.

Most mechanistic insights into the relationship between IGF-1 and brain function are derived from preclinical animal models. Experimental studies in aged rodents have demonstrated that exercise training can upregulate IGF-1 expression in the brain and activate the IGF-1/PI3K/Akt signaling pathway, leading to reduced apoptotic signaling, enhanced neuronal survival, and improved hippocampal function [89]. Similar effects have been observed in hypertensive rat models, where treadmill exercise increased cortical IGF-1/PI3K/Akt pathway activity and decreased the expression of pro-apoptotic factors [90]. These findings suggest that IGF-1–dependent signaling contributes to exercise-induced neuroprotection under pathological or aging-related conditions, although direct evidence in humans remains limited.

Preclinical evidence further indicates that increased IGF-1 signaling may promote synaptic plasticity and synapse formation. In rodent models, resistance training—but not aerobic exercise—has been shown to enhance hippocampal synaptic plasticity through the upregulation of the IGF-1/Akt pathway [91]. In addition, in a lipopolysaccharide-induced MCI rat model, a six-week resistance training intervention attenuated inflammatory responses in the dentate gyrus through activation of IGF-1 signaling, accompanied by improvements in cognitive performance [92]. These findings collectively support a role for IGF-1 in mediating exercise-related neuroplastic adaptations in animal models.

At the translational level, peripheral IGF-1 has been shown to cross the blood–brain barrier and enter the central nervous system [93]. Although circulating IGF-1 does not appear to alter hippocampal IGF-1 or IGF-1 receptor transcription directly, it can modulate the expression of genes involved in microvascular structure, brain development, and synaptic plasticity, which are critical for maintaining cognitive-related brain architecture [93]. Taken together, IGF-1 represents a biologically plausible mediator of exercise-induced brain benefits; however, further longitudinal and mechanistic studies in humans are required to clarify its causal role in cognitive aging.

Collectively, current evidence suggests that multiple myokines, including irisin, BDNF, IL-6, IL-15, and IGF-1, may contribute to exercise-related cognitive benefits through integrated muscle–brain signaling pathways. Rather than acting independently, these factors appear to form a coordinated network influencing neuroplasticity and neuroimmune balance. However, the magnitude and direction of their effects vary across physiological contexts and disease states.

Emerging studies also indicate that different exercise modalities elicit distinct myokine profiles, with resistance training being more consistently associated with IL-15 responses and aerobic exercise more strongly stimulating irisin secretion [94]. However, substantial heterogeneity in intervention protocols, participant characteristics, and biomarker assessment methods limits the ability to draw definitive conclusions. Future studies employing more standardized and mechanistically informed designs are required to clarify how exercise modality–specific myokine responses relate to cognitive outcomes in both healthy aging and neurodegenerative conditions.

## 6. Discussion

### 6.1. Synthesis of Evidence and Clinical Implications

The present review integrates evidence from randomized controlled trials, meta-analyses, and mechanistic studies to suggest that RT may serve as a viable non-pharmacological strategy to support cognitive function in older adults. Recent high-quality meta-analyses consistently report small to moderate improvements in global cognitive estimation and executive function following structured exercise interventions, with RT demonstrating particular promise compared with non-exercise controls or low-intensity activities [8,95,96]. While the magnitude of cognitive benefit varies across studies, the convergence of findings supports RT as a meaningful component of multimodal strategies aimed at preserving cognitive health during aging.

From a mechanistic perspective, RT appears to exert its cognitive effects through pathways that are distinct from, yet complementary to, those of aerobic exercise. Accumulating evidence supports the muscle–brain axis as a plausible biological framework, whereby RT-induced myokines—including BDNF, Irisin, IGF-1, and IL-6—may influence synaptic plasticity, neuroinflammation, and neurovascular function [9,47,50]. Notably, the current consensus emphasizes that preclinical and translational data primarily support these mechanisms, whereas direct causal evidence in humans remains limited. Therefore, RT-related cognitive improvements should be interpreted as biologically plausible and clinically relevant, but not yet mechanistically definitive.

### 6.2. Practical Recommendations, Safety, and Population-Specific Considerations

Translating current evidence into clinical guidance requires careful consideration of cognitive status, functional capacity, and the feasibility of interventions. Based on recent network meta-analyses and consensus statements, RT can be reasonably recommended for cognitively healthy older adults and individuals with MCI, particularly when programs incorporate progressive overload, moderate-to-high intensity, and adequate supervision [8,97]. In these populations, RT appears most effective for executive function, processing speed, and working memory, domains closely linked to functional independence.

In contrast, evidence in individuals with established dementia is more heterogeneous. While some small trials report modest short-term cognitive or behavioral benefits, larger pragmatic studies often show neutral effects, likely reflecting disease severity, limited task engagement, and reduced neuroplastic capacity [96]. Accordingly, RT in dementia populations should prioritize feasibility, safety, and quality of life outcomes rather than cognitive enhancement per se. For older adults with Parkinson’s disease, RT is generally supported for its motor and functional benefits, and may confer secondary cognitive advantages, particularly in executive domains; however, disease-specific evidence remains limited [98].

Safety considerations are central to clinical interpretation. Recent systematic reviews indicate that supervised RT is generally safe for older adults, including those with cognitive impairment, with a low incidence of serious adverse events such as falls or musculoskeletal injuries [98,99]. Reported challenges predominantly involve transient fatigue, muscle soreness, or adherence difficulties rather than intervention-related harm. Nevertheless, individuals with advanced cognitive impairment, frailty, or balance deficits may face increased risks if training complexity or intensity is not appropriately tailored. These findings support the use of individualized RT prescription, gradual progression, and close supervision as essential components of cognitively oriented exercise programs.

### 6.3. Limitations, Uncertainties, and Future Research Priorities

Despite encouraging findings, several limitations constrain current understanding of RT-induced cognitive benefits. First, the majority of mechanistic evidence relies on animal models or peripheral biomarker measurements, limiting causal inference regarding myokine-mediated effects on human brain function. Second, substantial heterogeneity in RT protocols, cognitive outcome measures, and participant characteristics precludes clear conclusions regarding optimal training dose, frequency, and modality. Third, adverse events, adherence, and feasibility outcomes are inconsistently reported, particularly in populations with dementia, reducing the translational applicability of existing trials.

Key uncertainties, therefore, remain regarding which cognitive domains are most responsive to RT, how long benefits persist after intervention cessation, and whether specific myokines directly mediate cognitive change. Future research should prioritize well-powered randomized controlled trials with standardized reporting of safety and feasibility outcomes, mechanistically informed biomarker panels, and longitudinal designs capable of testing mediation pathways. Integrating neuroimaging, circulating biomarkers, and cognitive assessments may further clarify how RT-induced muscular adaptations translate into brain structural and functional changes. Addressing these research priorities will be essential for refining evidence-based RT prescriptions tailored to different cognitive profiles in older adulthood.

## 7. Conclusions

This review highlights the RT as a potentially important exercise modality for supporting cognitive function in older adults, with emerging evidence suggesting a role for muscle–brain axis signaling. RT-induced myokines, including Irisin, BDNF, IL-6, IL-15, and IGF-1, may collectively contribute to neuroplasticity, neurovascular regulation, and inflammatory balance, thereby providing a biologically plausible framework linking skeletal muscle activity to cognitive outcomes. Compared with aerobic exercise, RT appears to elicit a distinct myokine response profile, indicating that RT may influence cognitive health through partially unique pathways.

Current evidence supports the use of structured and supervised RT as a feasible component of strategies aimed at preserving cognitive health in aging populations. However, the magnitude and consistency of cognitive benefits vary according to cognitive status, training parameters, and individual characteristics. Importantly, mechanistic evidence in humans remains limited, and causal relationships between RT-induced myokine responses and cognitive improvement have yet to be firmly established. Future research should, therefore, focus on clarifying dose–response relationships, delineating RT-specific mechanisms relative to other exercise modalities, and identifying individual factors that moderate responsiveness to RT. Advancing this line of research will be essential for developing evidence-based, muscle–brain axis-informed exercise interventions to promote cognitive resilience in older adulthood.

## Figures and Tables

**Table 1:** Comparison of RT intervention parameters and cognitive improvement effects in elderly populations with different cognitive states.

Author (Year)	Participant Characteristics	Exercise Intervention Protocol	Cognitive Assessment Methods and Intervention Outcomes	Randomization Method	Blinding	Primary Outcomes	Follow-Up Duration	Effect Sizes (Cohen’s d or SMD)	Risk of Bias Indicators
Maren S. Fragala (2014) [17]	Healthy older adults *(n* = 25, aged 64–76), randomly assigned to RT or Control (CON).	RT Group (*n* = 13): Whole-body RT, moderate intensity (RPE 5–6), three sets of 8–15 reps, twice weekly for 6 weeks. CON Group (*n* = 12): No intervention.	Assessments: Neurotracker, Dynavision. Results: The RT group showed significantly greater improvements in spatial awareness (+40.0% vs. +2.9%) and visual reaction time (+14.6% vs. +1.7%) compared to the CON group.	Computerized randomization	Single-blind	Spatial awareness, visual reaction time	6 weeks	Spatial awareness: Cohen’s d = 0.85 Visual reaction time: Cohen’s d = 0.92	Low risk of bias
Carla Coetsee (2017) [18]	Healthy older adults (*n* = 67, aged 55–75), randomly assigned to RT, HIIT, MCT, or CON.	RT Group (*n* = 22): Machine/free-weight RT, 3 × 10 reps (50–100% 10RM). HIIT (*n* = 13), MCT (*n* = 13): Aerobic protocols. CON Group (*n* = 19): No intervention.	Assessment: Stroop task. Results: RT significantly improved executive function (Stroop conflict) compared to HIIT, MCT, and CON, with stable task accuracy.	Stratified randomization	Double-blind	Executive function (Stroop conflict)	16 weeks	Cohen’s d = 1.10 (RT vs. CON)	Moderate risk of bias
Nils Eckardt (2020) [19]	Healthy older adults (*n* = 68, aged 65–79), randomly assigned to Unstable Free-Weight RT (I-FRT), Stable Machine-Based RT (S-MRT), or Stable Machine-Based Hip RT (S-MRT HIP).	I-FRT (*n* = 21): Free-weight exercises with unstable devices. S-MRT (*n* = 24): Machine-based exercises. S-MRT HIP (*n* = 23): Hip adduction/abduction. All groups trained twice weekly for 10 weeks.	Assessments: DST, DSST, SCWT, TMT. Results: The I-FRT group showed significant within-group improvements in working memory, processing speed, and response inhibition, outperforming stable groups.	Block randomization	Double-blind	Working memory, processing speed, and response inhibition	10 weeks	Working memory: Cohen’s d = 0.32 Processing speed: Cohen’s d = 0.73	Low risk of bias
Kathryn M. Broadhouse (2020) [20]	Older adults with MCI (*n* = 100, aged 62–76), randomly assigned to PRT + computerized cognitive training (CCT), PRT + sham CCT, CCT + sham RT, or double-sham control.	Progressive resistance training (PRT): pneumatic resistance machines, 3 sets × 8 repetitions, 2–3 sessions/week for 26 weeks; applied to relevant groups	Assessments: ADAS-Cog, executive function tests, hippocampal subfield volumetry (MRI). Results: PRT alone (PRT + sham CCT) improved global cognition and executive function and attenuated hippocampal subfield atrophy at long-term follow-up; combined PRT + CCT showed no additional benefit	Computer-generated randomization	Single-blind (outcome assessors)	Global cognition (ADAS-Cog); executive function; hippocampal subfield volume	26-week intervention; 12-month and 18-month follow-up	NR (standardized effect sizes not reported)	Low–moderate risk (randomized design; multiple arms; limited reporting of effect sizes)
Ji-Eun Baek (2024) [21]	Older adults with MCI (*n* = 44, aged 76–86), randomly assigned to dual-task resistance exercise (DTRE) or single-task resistance exercise (STRE)	DTRE: resistance exercise combined with concurrent cognitive tasks; STRE: resistance exercise alone. Protocol: 3 sets × 10 repetitions, 3 sessions/week for 6 weeks	Assessment: MMSE-K. Results: Both groups showed post-intervention cognitive improvement, with significantly greater MMSE gains in the DTRE group compared with STRE	Random allocation (method not specified)	Single-blind (assessors)	Global cognitive function (MMSE-K)	6 weeks	NR (standardized effect sizes not reported)	Moderate risk (short intervention duration; randomization method not fully specified)
Wouter A. J. Vints (2024) [22]	Older adults at high risk of MCI (*n* = 52, aged 60–85), randomly assigned to RT or control	RT: machine-based resistance training (e.g., leg press), 2–3 sets × 6–10 repetitions, twice weekly for 12 weeks; control: no intervention	Assessments: Go/No-Go task, mathematical processing task, serum biomarkers (IGF-1). Results: RT improved inhibitory control (reduced Go/No-Go reaction time); increases in IGF-1 were associated with faster processing speed	Block randomization	Single-blind (outcome assessors)	Inhibitory control, processing speed	12 weeks	Effect size reported as correlation (IGF-1 vs. processing speed: r = −0.497)	Low risk (randomized design; assessor blinding)
I-Ting Liu (2020) [23]	Older adults with dementia (*n* = 61, aged 66–95), randomly assigned to RT or aerobic training (AT)	RT group (*n* = 30): upper- and lower-limb isotonic resistance training at 40–50% 1RM, 2 × 12 repetitions; AT group (*n* = 31): stationary cycling. Both groups trained 5 sessions/week for 4 weeks	Assessments: MMSE, MoCA; plasma inflammatory biomarkers. Results: Both RT and AT significantly improved MMSE scores, with no significant between-group difference. RT was associated with a reduction in plasma MCP-1 levels	Random allocation (method not specified)	Single-blind (outcome assessors)	Global cognitive function (MMSE, MoCA); inflammatory marker (MCP-1)	4 weeks	NR (standardized effect sizes not reported)	Moderate risk (short intervention duration; limited reporting of randomization procedure)
Sarah E. Lamb (2018) [24]	Older adults with mild-to-moderate dementia (*n* = 494, aged 69–84), randomly assigned to exercise intervention or usual care control	Exercise group (*n* = 329): combined moderate-to-high intensity aerobic and resistance training, twice weekly for 4 months; control group (*n* = 165): usual care	Assessment: ADAS-Cog. Results: The exercise intervention was associated with a modest worsening of cognitive performance compared with usual care (between-group difference: −1.4 points; 95% CI −2.6 to −0.2; *p* = 0.03)	Computer-generated randomization	Assessor-blinded	Global cognitive function (ADAS-Cog)	4 months	NR (standardized effect sizes not reported)	Low risk (large sample size; randomized multicenter design; assessor blinding)
Fabian J. David (2015) [25]	Older adults with mild-to-moderate PD (*n* = 48, aged 50–67), randomly assigned to Progressive Resistance Exercise Training (PRET) or modified Fitness Counts (mFC)	PRET (*n* = 24): machine-based RT with progressive load; mFC (*n* = 24): stretching, balance, and non-progressive strength exercises; both groups trained twice weekly for 24 months	Assessments: Digit Span, Stroop Test, and Brief Test of Attention. Results: PRET led to significant improvements in working memory, inhibitory control, and selective attention compared with mFC	Random allocation (method not specified)	NR	Cognitive performance (working memory, attention, inhibition)	24 months	NR (standardized effect sizes not reported)	Moderate risk (long-term RCT; randomization and blinding procedures incompletely reported)
Carla Silva-Batista (2016) [26]	Older adults with moderate PD and MCI (*n* = 39, aged 50–80), randomly assigned to Unstable RT (RTI), Stable RT (RT), or Control	RTI (*n* = 13): machine-based RT on unstable surfaces; RT (*n* = 13): machine-based RT on stable surfaces; Control (*n* = 13): health education; RT protocols: 2–4 sets of 6–12 repetitions, twice weekly for 12 weeks	Assessments: MoCA and domain-specific cognitive tests. Results: RTI significantly improved global cognition (MoCA) and showed superior gains in executive function and attention compared with RT and control	Random allocation (method not specified)	Single-blind (outcome assessors)	Global cognition (MoCA), executive function, and attention	12 weeks	NR (standardized effect sizes not reported)	Moderate risk (small sample size; short intervention duration)
Carla Silva-Batista (2025) [27]	Older adults with moderate-to-severe PD, freezing of gait (FOG), and MCI (*n* = 32, aged 49–85), randomly assigned to Adapted RT with Instability (ARTI) or Traditional Motor Rehabilitation (TMR)	ARTI (*n* = 17): complex free-weight exercises using unstable devices; TMR (*n* = 15): stretching, gait and balance training, and stable RT; both groups trained 3 sessions/week for 12 weeks	Assessments: Frontal Assessment Battery (FAB), MoCA, Digit Symbol Substitution Test (DSST). Results: ARTI significantly improved frontal lobe function, global cognition, and attention. Improvements in frontal function were significantly associated with reductions in FOG severity	Random allocation (method not specified)	Single-blind (outcome assessors)	Frontal executive function; global cognition; attention	12 weeks	NR (standardized effect sizes not reported)	Moderate risk (small sample size; limited reporting of allocation concealment)

**Table 2:** Cross-sectional epidemiologic studies examining correlations between muscle mass or function and cognitive indicators.

Participant Characteristics	Correlation Findings
2540 older adults (43.3% male); aged ≥ 60 years; mean age: 70.43 years [35]	Low muscle mass and cognitive dysfunction were significantly correlated in the older population (*p* < 0.001).
>40,000 participants (49% male); aged ≥ 45 years; mean age: 64.21 years [36]	Grip strength was significantly correlated with faster reaction time (N = 40,278, t = −19.56, FDR-corrected *p* = 2.42 × 10^−83^). Positive correlations were also observed with other cognitive tasks (e.g., fluid intelligence, prospective memory).
245 older adults (45.3% male); aged ≥ 60 years; mean age: 76.22 years [37]	Grip strength was significantly positively correlated with 1-back accuracy rate (r = 0.342, *p* < 0.001); The 30 s chair stand test was significantly positively correlated with 1-back accuracy rate (r = 0.616, *p* < 0.001).
1723 older adults (42.77% male); aged ≥ 60 years; mean age: 68.52 years [38]	Each 1 kg increase in grip strength was associated with a 3.5% reduction in cognitive impairment risk (OR: 0.965; 95% CI: 0.947–0.984; *p* < 0.001). Cognitive impairment was also significantly associated with decreased grip strength (OR: 0.264; 95% CI: 0.119–0.583; *p* < 0.001).
595 older adults (40.8% male); aged ≥ 65 years [39]	Lower limb muscle strength was significantly positively correlated with cognitive function (r = 0.286, *p* < 0.01).
109 older adults (46.8% male); aged ≥ 70 years; mean age: 80.10 years [40]	Physical activity level was significantly positively correlated with grip strength (r = 0.559, *p* < 0.001); Grip strength was significantly positively correlated with 1-back accuracy rate (r = 0.417, *p* < 0.001); Grip strength was significantly negatively correlated with 0-back reaction time (r = −0.478) and 1-back reaction time (r = −0.441) (all *p* < 0.001).

## Data Availability

No new data were generated or analyzed in this study. Data sharing does not apply to this article.
